# Late surgical treatment of tetralogy of Fallot

**DOI:** 10.5830/CVJA-2010-057

**Published:** 2011-08

**Authors:** JCT Tchoumi, JC Ambassa, A Giamberti, S Cirri, A Frigiola, G Butera

**Affiliations:** St Elizabeth Catholic General Hospital, Cardiac Centre, Shisong, Cameroon; St Elizabeth Catholic General Hospital, Cardiac Centre, Shisong, Cameroon; Policlinico San Donato IRCCS, Pediatric Cardiology, Cardiac Surgery and GUCH Unit, Milan, Italy; Policlinico San Donato IRCCS, Pediatric Cardiology, Cardiac Surgery and GUCH Unit, Milan, Italy; Policlinico San Donato IRCCS, Pediatric Cardiology, Cardiac Surgery and GUCH Unit, Milan, Italy; Policlinico San Donato IRCCS, Pediatric Cardiology, Cardiac Surgery and GUCH Unit, Milan, Italy

**Keywords:** tetralogy of Fallot, older patients, follow up

## Abstract

**Aim:**

To study early post-operative results and follow up of patients over a year old, operated on for tetralogy of Fallot (ToF).

**Methods:**

This retrospective analysis included 22 patients (14 male and eight female) with a mean age of 9.18 ± 6.5 years (range 13.5 months to 26 years), who underwent complete repair of ToF between April 2003 and June 2009. Data from patients’ records, pre-operative cardiac catheterisation studies, operative intervention, and pre-operative and postoperative two-dimensional echocardiographic studies were reviewed. All patients underwent complete repair including closure of ventricular septal defect (VSD). A trans-annular patch was used in 12 patients while an infundibular patch was used in 10 others. Patients were evaluated one, three, six and 12 months after surgery, and annually thereafter. The duration of follow up was from eight months to six years post surgery.

**Results:**

Classical ToF was found in 10 patients. Twelve cases had associated anomalies: two patients with hypoplastic pulmonary artery branches, two with arterial duct malformations, and eight had proximal stenosis of the left branch of the pulmonary artery. NHYA class distribution was as follows: class I: two patients; class II: five subjects; class III: 10 patients; class IV: five subjects. The mean stay in hospital was 15 ± 7 days. Two patients (9%) died during the early post-surgical period. At a mean follow-up interval of 32 ± 9 months, all patients were asymptomatic and in NYHA class I. No late deaths occurred. In three patients, we registered isolated monomorphic ventricular extrasystoles. The right ventricle outflow tract (RVOT) pressure gradient was 29 ± 1.5 mmHg in the acute post-surgical period and it did not change significantly during follow up. The right ventricular function was defined as normal in 95% of the patients in the study and was mildly depressed in 5%.

**Conclusion:**

Even if treated later in life, our study showed very good surgical results of patients with ToF.

## Abstract

Tetralogy of Fallot (ToF) is the most common cause of cyanotic congenital heart disease.^1^ The best age for ToF repair remains controversial and the strategy employed may influence the technique of operation.^2^ Early repair of ToF has many advantages. It abolishes the secondary effects of increasing cyanosis on vital organs and its adverse effects on cognitive and psychomotor development of the patient. The relief of right ventricular outflow obstruction eliminates the secondary right ventricular hypertrophy and maintains the systolic and diastolic properties of the ventricle when compared with either two-stage repair or repair later in life.^3^ Supporters of primary neonatal repair cite factors such as prevention of time-related end-organ damage from cyanosis, removal of stimulus for right ventricular hypertrophy and fibrosis, improved lung development (vascular and alveolar), avoidance of deleterious effects and risks of palliative shunts, and psychosocial-economic issues (for the family and care givers).^4^

In large centres worldwide, correction is performed between six and nine months of age.^5^ However, in developing countries, early repair may be difficult due to many factors, including facilities for diagnosis, cultural background of patients, and availability of surgical centres. The purpose of this retrospective investigation was to study the early results and follow up in patients with ToF operated on when older than a year and coming from a developing country.

## Methods

This retrospective analysis included 22 patients (14 males and eight females) with a mean age of 9.18 ± 6.5 years (range 13.5 months to 26 years) who underwent complete repair of ToF between April 2003 and June 2009. All subjects came from Cameroon and were evaluated in the Cardiac Centre of the Saint Elizabeth Hospital in Shisong. Data from patients’ records, pre-operative cardiac catheterisation studies, operative intervention, and pre- and postoperative two-dimensional echocardiographic studies were reviewed.

All patients were operated on in the paediatric surgical unit of San Donato teaching hospital in Shisong, and the project was supported by the Tertiary Sisters of St Francis and two nongovernmental, non-profit organisations, Cuore Fratello (www.cuorefratello.org) and Associazione Bambini Cardiopatici nel Mondo (www.bambinicardiopatici.it).

The ethics committee of the hospital approved the study. A consent form was signed by patients, or their parents when patients were unable to sign. It is significant that during the period of the study while awaiting surgery, 25 patients aged between seven months and 15 years (nine males) died due to ToF-related complications.

The operative technique to repair ToF has been described previously.^6^ RVOT reconstruction included a trans-annular patch in the pericardium with a monocusp made of goretex. Some subjects needed a trans-infundibular patch without any surgical treatment of the pulmonary valve.^6^

Patients were evaluated at one, three, six and 12 months, and then annually after surgery. Postoperative evaluation included a complete physical examination, an electrocardiogram, and two-dimensional and Doppler echocardiography in all patients.

## Statistical analysis

Statistical analyses were performed using the χ^2^ test and the Wilcoxon rank sum test for non-parametric variables. A paired *t*-test was used for continuous variables. The results were calculated as the mean ± standard deviation. All statistical analyses were performed using the SPSS 11 program.

## Results

Classical ToF was found in 10 patients. Twelve cases had associated anomalies (two patients with hypoplastic pulmonary artery branches, two with arterial duct malformations, and eight had proximal stenosis of the left branch of the pulmonary artery). NYHA class distribution was as follows: class I: two patients; class II: five subjects; class III: 10 patients; class IV: five subjects.

Five patients (25%) were symptomatic, with more than one episode of hypoxic spells or persistent hypoxaemia (arterial oxygen saturation less than 80%). Among these was one boy with hypotony of the lower limbs because of prolonged immobility due to the hypoxic spells during mild physical exertion.

## Early postoperative course

Complete repair was carried out on 22 patients. Closure of the ventricular septal defect (VSD) with the insertion of a transannular patch was performed in 12 subjects, and closure of the VSD with the insertion of an infundibular patch was performed in 10 patients. The acute post-surgical complications were mild pericardial effusion detected in four cases and pleural effusion in three cases. The dosage of diuretic was increased in these, with good results. In one case there was chylothorax, which was treated with diet. There were no cases of arrhythmia. We did not observe any differences in follow-up results regarding age of the children at surgery, nor for surgical technique.

The mean hospital stay was 15 ± 7 days and the range was between 10 and 20 days. In the intensive care unit, the mean stay was 5 ± 3 days. The chest tubes for drainage were removed in the general children’s ward, mostly on the seventh to eighth postoperative day. The mean oxygen saturation of the patients changed from 67 ± 5% before surgery to 90 ± 2.5% after the correction.

A five-year-old boy and a girl died in the acute post-surgical period (9%). The cause of death was profuse bleeding from a poorly controlled collateral vessel.

## Follow-up-data

The duration of follow up was from eight months to six years. *Electrocardiographic data*: All patients were in regular sinus rhythm. First-degree atrio-ventricular block was observed in two patients in the acute post-surgical period and thereafter. Right bundle branch block and right ventricular hypertrophy present before surgery persisted in 88% of patients. In three patients we detected isolated monomorphic ventricular extra-systoles. *Echocardiographic data*: There were no significant differences in RVOT gradients and right ventricular (RV) pressures during follow up in all patients except one, who developed mild to moderate RVOT obstruction, with a pressure gradient of 25 to 35 mmHg proximal to the patch. The mean RVOT pressure gradient was 29 ± 1.5 mmHg in the acute post-surgical period. Three months later, it was 27.6 ± 1.7 mmHg. The RVOT pressure at one year was 26.8 ± 1.1 mmHg (*p* ≤ 0.05) compared to the acute post-surgical reading. After two years, the mean RVOT pressure was constant at around 26.2 ± 2.9 mmHg (*p* > 0.6). This was maintained for about two years, until the last follow-up measurements.

Right ventricular function was defined as normal in 95% of the patients in the entire study and mildly depressed in 5%. A mild to moderate degree of pulmonary regurgitation was evident in three patients, associated with mild triscupid regurgitation and a mild right ventricular dilatation.

A diagnostic cardiac catheterisation was done prior to surgery in only three cases, for better visualisation of the coronary arteries, pulmonary arterial trunk and pulmonary branches. Proximal left pulmonary artery stenosis with a pressure gradient of 20 mmHg was found in one patient a week after surgery. A successful dilatation was done in the catheterisation laboratory.

## Discussion

Although the presentation of ToF in adults is becoming a rarity in Western countries, it is still common in developing countries. In our study we aimed to analyse the early results and follow up of older Cameroonian patients operated on for ToF. All our patients had surgery after 12 months of age, with a good outcome.

In the literature, correction is usually performed before one year of age and some authors advocate very early repair, before six months of age. However, Vohra *et al*.^5^ found that early primary repair of ToF was comparable to later repair. Ooi *et al*.^7^ showed that early definitive repair of ToF can be performed safely on patients under six months old. Both approaches are supported by clinical reports of excellent early results.^8^ However, in our cases, because of lack of adequate medical facilities and poverty of patients, the diagnosis and surgery were performed late.

In our cohort of ToF patients, the most commonly occurring problems before surgery were hypotony of the lower limbs and stiffness caused by immobility due to hypoxic spells. After surgery, we observed pulmonary and triscupid regurgitation and residual obstruction of the RVOT. It is important to mention that hypoxic spells during mild physical exertion are an incapacitating factor in our context, leading to stiffness and hypotony of the lower limb muscles, which will need later physiotherapy.

Late diagnosis and late surgical intervention are probably the reasons why early mortality in our series was 9%, which is quite elevated when compared to data by Wu^9^ (0.9% death rate in patients aged between one and 37 years) or Ghavidel *et al*.^10^ (1.9% in adult patients with ToF). In our case, the cause of death was poorly controlled severe bleeding from the collaterals. It is well known that deeply cyanosed patients have a well-developed collateral circulation. Furthermore, the presence of combined chronic latent diseases (tropical infections) on admission could have influenced the acute post-surgical period.

The length of the chest tube for drainage was notably longer in our series than in ToF subjects treated between six and 12 months of age. Furthermore, post surgery, hypotony of the lower limbs was present in one patient who had a longer hospital stay to enable the lower limbs to recover their function. These factors accounted for the longer length of hospital stay in our series.

During follow up, no deaths were registered. All patients were in NYHA class I. Similar data were reported by Bisoi *et al*.,^11^ who stated that total correction of ToF in teenagers and adults offers the best option for long-term, symptom-free survival.

No malignant rhythm disturbances were recorded on ECG. Right ventricular hypertrophy with a right bundle branch block (RBBB) recorded on ECG persisted after surgery. Bouzas^12^ showed that the presence of RBBB in post-TOF repair is extremely common and may mask the presence of RV hypertrophy. Progressive lengthening of the QRS duration with time reflects RV enlargement and potential RV failure.

We observed a drop, although not statistically significant, in the RVOT pressure from the acute post-surgical period. No difference in RVOT pressure was registered between the patients with annuloplasty and infundibuloplasty. No restrictive Doppler pattern was observed in assessing the right ventricle, contrary to the study of Cardoso and Miyague,^13^ who reported that restrictive right ventricular physiology was present in most patients undergoing repair of ToF with a trans-annular patch. This may be a transient phenomenon of incomplete adaptation of the right ventricle to volume and pressure modifications.

Over six years of follow up, right ventricular function was mildly depressed in 5% of cases, and mild to moderate pulmonary regurgitation was evident in 13.7%. Pulmonary regurgitation (PR) is related to the use of a trans-annular patch during RVOT reconstruction and aggressive infundibulectomy involving the pulmonary valve annulus. Although PR is reportedly well tolerated in several clinical studies, long-term follow up has shown that this can lead to considerable disability. The adverse effects of PR include progressive dilatation of the RV, reduced exercise capacity, arrhythmia and sudden death. The right ventricular function and pulmonary valve of these patients will need close monitoring to anticipate pulmonary or tricuspid valve replacement.

Our study had two limitations. First, we had a limited number of patients enrolled. Second, longer follow up is needed.

## Conclusion

The study showed good early results in the post-surgical follow up of older patients with ToF. The right ventricular function and pulmonary valve will need close surveillance, and timely and appropriate interventions must be taken to optimise outcomes.
